# *Populus alba* cationic cell-wall-bound peroxidase (CWPO-C) regulates the plant growth and affects auxin concentration in *Arabidopsis thaliana*

**DOI:** 10.1007/s12298-022-01241-0

**Published:** 2022-10-30

**Authors:** Diego Alonso Yoshikay-Benitez, Yusuke Yokoyama, Kaori Ohira, Koki Fujita, Azusa Tomiie, Yoshio Kijidani, Jun Shigeto, Yuji Tsutsumi

**Affiliations:** 1 Department of Agro-environmental Sciences, Graduate School of Agriculture, Kyushu University, 744 Motooka Nishi-ku, Fukuoka, 819-0395 Japan; 2 Faculty of Agriculture, Kyushu University, 744 Motooka Nishi-ku, Fukuoka, 819-0395 Japan; 3 Division of Forest and Environmental Science, Faculty of Agriculture, University of Miyazaki, 1-1 Gakuen Kibana-dai Nishi, Miyazaki, 889-2192 Japan; 4 Office of Research and Academia Government Community Collaboration, Hiroshima University, 1-3-2 Kagamiyama, Higashihiroshima, Hiroshima 739-8511 Japan

**Keywords:** *Arabidopsis thaliana*, CWPO-C, Gravitropism, IAA, Overexpression, Plant peroxidase

## Abstract

The poplar cationic cell-wall-bound peroxidase (CWPO-C) mediates the oxidative polymerization of lignin precursors, especially sinapyl alcohols, and high molecular weight compounds that cannot be oxidized by other plant peroxidases, including horseradish peroxidase C. Therefore, CWPO-C is believed to be a lignification-specific peroxidase, but direct evidence of its function is lacking. Thus, the *CWPO-C* expression pattern in *Arabidopsis thaliana* (Arabidopsis) was determined using the *β*-glucuronidase gene as a reporter. Our data indicated that *CWPO-C*  was expressed in young organs, including the meristem, leaf, root, flower, and young xylem in the upper part of the stem. Compared with the wild-type control, transgenic Arabidopsis plants overexpressing *CWPO-C* had shorter stems. Approximately 60% of the plants in the transgenic line with the highest CWPO-C content had curled stems. These results indicate that CWPO-C plays a role in cell elongation. When plants were placed horizontally, induced *CWPO-C* expression was detected in the curved part of the stem during the gravitropic response. The stem curvature associated with gravitropism is controlled by auxin localization. The time needed for Arabidopsis plants overexpressing *CWPO-C* placed horizontally to bend by 90° was almost double the time required for the similarly treated wild-type controls. Moreover, the auxin content was significantly lower in the *CWPO-C*-overexpressing plants than in the wild-type plants. These results strongly suggest that CWPO-C has pleiotropic effects on plant growth and indole-3-acetic acid (IAA) accumulation. These effects may be mediated by altered IAA concentration due to oxidation.

**Supplementary Information:**

The online version contains supplementary material available at 10.1007/s12298-022-01241-0.

## Introduction

Plant peroxidases (EC 1.11.17, Class III peroxidases; Prxs) are plant-specific heme oxidoreductases comprising approximately 300 amino acid residues. Land plants contain many genes encoding Prxs localized in the apoplast or vacuole (Zipor et al. 2013). Peroxidases catalyze reactions affecting several important physiological and developmental processes (e.g., lignification, stress responses, development, and germination), with diverse substrate specificities and reactivities. However, the function and the substrate of most Prxs remain unidentified. Thus, the available information regarding the mechanism underlying the in vivo activity and role of Prxs is limited. Regarding lignin polymerization (i.e., lignification), a repeating radical coupling of a new monomer to the growing polymer (or oligomer) in the cell wall occurs via oxidative coupling. Lignin is composed of three major monolignols, namely *p*-coumaryl alcohol, coniferyl alcohol, and sinapyl alcohol. This oxidative coupling mechanism is catalyzed by Prxs and/or laccase.

Peroxidases can catalyze the oxidation of a broad range of substrates. The well-studied Prxs Horseradish peroxidase isoenzyme C (HRP-C) and AtPrx53 minimally oxidize sinapyl alcohol and the lignin oligomer because these potential substrates are larger than the heme pocket of these two enzymes. However, we previously reported that the cationic cell-wall-bound Prx (CWPO-C) from *Populus alba* can efficiently oxidize lignin monomers, including sinapyl alcohol, and high molecular weight lignin polymers (Aoyama et al. 2002; Sasaki et al. 2004). This unique oxidation is facilitated by the fact that the oxidation sites (Tyr74 and Tyr177) of CWPO-C are located on the protein surface, enabling the enzyme to avoid steric hindrance during enzyme–substrate interactions (Shigeto et al. 2012). This finding suggests that the formation of lignin containing sinapyl alcohol in the cell wall can be catalyzed by a certain type of Prx, including CWPO-C. Furthermore, we reported that the putative *Arabidopsis thaliana* (Arabidopsis) homologs of CWPO-C, AtPrx2, AtPrx25, and AtPrx71 (48%, 66%, and 68% amino acid identity with CWPO-C, respectively), can also oxidize sinapyl alcohol and lignin polymers (Shigeto et al. 2014). Silencing *AtPrx2*, *AtPrx25*, and *AtPrx71* affects lignin-related phenotypes by decreasing the lignin content and/or changing the lignin structure (Shigeto et al. 2013, 2015).

The results of earlier studies suggested that CWPO-C is responsible for the lignification process in stem. In this study, we further characterized the possible in vivo function of CWPO-C on the basis of a spatiotemporal analysis of gene expression using transgenic plants expressing *β*-glucuronidase (GUS) under the control of the *CWPO-C* promoter as well as a phenotypic analysis of *CWPO-C*-overexpressing (OE) Arabidopsis lines. Unexpectedly, our findings revealed that CWPO-C has functions associated with plant growth. Phenotypic observations of *CWPO-C* overexpressed Arabidopsis plants revealed alterations in plant growth and tropism alterations which coincide with the indole-3-acetic acid (IAA) deficient phenotype. The liquid chromatography–tandem mass spectrometry (LC-MS/MS) analyses showed remarkable decrease in IAA content in the transgenic plant. This finding can be explained by the in vitro catabolism of IAA by peroxidases (Galston et al. 1953; Hinman and Lang 1965), and results in this study strongly suggested that CWPO-C contributes to the primary plant growth stage and tropism via IAA catabolism.

## Materials and methods

### Plant materials and growth conditions

Wild-type (WT) and transgenic *Arabidopsis thaliana* (ecotype Columbia) plants were used in this study. Seeds were immersed in 0.5% (v/v) sodium hypochlorite for 5 min and then rinsed three times with sterile water. After imbibition at 4 °C for 1 day, the seeds were sown in plastic Petri dishes containing Murashige and Skoog (MS) medium supplemented with 3% (w/v) sucrose, pH 5.6, and 0.3% (w/v) gellan gum. The plates were incubated in CLE-303 cultivation chambers (TOMY SEIKO Co., Ltd., Tokyo, Japan) set at 22 ± 1 °C with a 16-h light (100 µmol photons m^− 2^ s^− 1^):8-h dark cycle. Three-week-old seedlings were transferred to pots containing vermiculite and perlite (1:1, v/v) and were subsequently irrigated with 0.1% Hyponex (Hyponex Japan, Osaka, Japan) every 4 days.

### Generation of transgenic plants

The transgenic plants used in this study were Arabidopsis ecotype Columbia background. Complete *CWPO-C* sequence and *CWPO-C* promoter sequence were previously prepared from *Populus alba* (Sasaki et al. 2006). To generate a fusion construct comprising the *CWPO-C* promoter and the *GUS* marker gene (*Pcwpo-c*::*GUS*) (Fig. S3a) and a *CWPO-C* overexpression construct consisting of the CaMV 35S promoter (*P35S*::*CWPO-C*) (Fig. S3b), the 1,855-bp 5′ upstream region of *CWPO-C* and the complete *CWPO-C* open reading frame were amplified by PCR and then subcloned into the pBluescriptII vector (Qiagen). The PCR primer sequences were as follows: *P*_*CWPO−C*_-left (5′-gcgcaagcttagggaaacaactaaaataccattaataac-3′) and *P*_*CWPO−C*_-right (5′-gttggatcctgtatatgctagctaagagactg-3′); *CWPO-C*-left (5′-cgggatccatgagccaaaaagtagttttaatgtttc-3′) and *CWPO-C*-right (5′-ccgagctcatttatagcagaacacactcttcgaatttcac-3′). For the *Pcwpo-c*::*GUS* fusion construct, the 1,855-bp 5′ upstream region of *CWPO-C* and the *GUS* gene were introduced between *Hind*III and *Bam*HI and between *Bam*HI and *Sac*I, respectively, in the binary vector PBF2 (Nishiguchi et al. 2006). Regarding the *CWPO-C* overexpression construct, the complete *CWPO-C* open reading frame (Sasaki et al. 2006) was introduced between *Bam*HI and *Sac*I in the PBF2 vector. For each construct, the binary vector was inserted into *Agrobacterium tumefaciens* strain LBA4404 cells, which were then used to transform plants according to the floral dip method (Clough and Bent 1998). The transgenic plants were selected on half-strength MS medium supplemented with 50 mg l^− 1^ kanamycin. Seeds were collected from each kanamycin-resistant F_1_ plant. Homozygous T_2_ plants were identified and selected according to the proportion of kanamycin-resistant plants. Homozygous T_3_ and T_4_ transgenic plants were used for further analyses.

### Western blot analysis

Whole aerial plant parts without roots from 2-week-old WT and transgenic Arabidopsis seedlings, grown in Petri dishes containing MS medium were collected and frozen in liquid nitrogen. Later, the ground powder was mixed with 3 equivalent volumes (w/v; mg µl^−1^) of sodium dodecyl sulfate (SDS) sample buffer containing 50 mM Tris-HCl (pH 7.4), 2% SDS (w/v), 6% β-mercaptoethanol (v/v), 10% glycerol (v/v), and 0.001% bromophenol blue and then homogenized. The homogenate was centrifuged at 20,000 *g* for 20 min. The protein content in the supernatant was determined using the Pierce 660 nm Protein Assay kit (Thermo Fisher Scientific).

A 10-µg protein sample was separated by 10% (w/v) SDS polyacrylamide gel electrophoresis and then electroblotted onto a PVDF membrane (Thermo Fisher Scientific). To confirm the equal loading of protein, an identical gel was prepared and stained in parallel with Coomassie brilliant blue. The CWPO-C protein was detected using an anti-CWPO-C antibody and the anti-rabbit IgG secondary antibody conjugated with horseradish Prx (Proteintech Group Inc.) at a dilution of 1:500 (v/v) and 1:2000 (v/v) in phosphate buffer (pH 7.4), respectively. The anti-CWPO-C antibody was purified from the serum of rabbits immunized with purified recombinant CWPO-C that was prepared by GENE NET (Fukuoka, Japan) as described by Shigeto et al. (2012). The antigen–antibody complex was visualized using AE-1490 EzWestBlue (ATTO).

### GUS staining

Transgenic plants were analyzed for GUS staining using a modified version of a published procedure (Jefferson et al. 1987). Briefly, seedlings were treated with 90% cold acetone and incubated for 1 h at − 20 °C to remove chlorophyll. After washing twice with 100 mM phosphate buffer (pH 7.4), the seedlings were transferred to the GUS staining solution containing 100 mM NaPO_4_ (pH 7.4), 0.5 mM 5-bromo-4-chloro-3-indolyl-β-D-glucuronide, 2.5 mM K_3_[Fe(CN)_6_], and 2.5 mM K_4_[Fe(CN)_6_]. After  vacuum infiltration at − 0.6 MPa, the seedlings were incubated at 37 °C for 14 to 16 h. The seedlings were washed with the following solutions: 70% ethanol for 30 min, an ethanol:acetic acid solution (9:1, v/v) for 2 h, 90% ethanol twice for 30 min each, and a chloral hydrate:water:glycerol solution (25:9:3, w/v/v) for 4 to 12 h. Stem cross-sections of 15 μm thickness were prepared by cryosectioning using the Tissue-Tek® OCT compound (Sakura-Finetek).

### Histochemical assay

Cell wall lignin was detected in the stem sections of WT and transgenic plants using the Wiesner staining. Plants collected at 5-week-old, and 25 μm cross sections were made from the 0.5 cm of the basal parts of stems. The sections were prepared by the cryo-sectioning, and cryo-matrix compound was removed with water. Sections were stained with1% (w/v) phloroglucinol for 3 min by Wiesner staining as described by Euring et al. (2012). Three biological replicates for each WT and OE11 were used for phloroglucinol staining. At least 2 cross sections per plant were observed by the microscope. Optical microscopic observation was conducted with Keyence VHX-6000 optical microscopy.

### In vitro reaction of IAA with recombinant CWPO-C and HPLC analysis

Recombinant CWPO-C (rCWPO-C) was prepared and purified according to the described methods by Shigeto et al. (2012). The oxidation of IAA by rCWPO-C was carried out in a 100 mM sodium acetate buffer (pH 5) containing 500 µM H_2_O_2_, 500 µM IAA, 2 U/ml rCWPO-C and rotary-incubated (Rotaflex, Argos) at 10 rpm for 0, 60, 120, 360 min at 25 °C. One U of CWPO-C oxidation activity was defined as the formation of the oxidation product from 2,6-dimethoxyphenol (2,6-DMP) in one µmol/min (Shigeto et al. 2012). As control, the reaction was carried out in the absence of rCWPO-C. Reaction mix was filtered with a Minisart SRP 4 PTFE-membrane and an aliquot of reaction mixture (20 µl) was analyzed by reverse-phase HPLC on a InertSustain AQ-C_18_ column (100 nm, 5 μm, 4.6 × 150 mm; GL Sciences, Japan) using an isocratic elution buffer containing methanol : 1% formic acid mixture (40:60, v/v) at a flow rate of 1.0 ml/min. The eluted products were monitored at the absorbance of 250 nm using a Jasco UV-2070 Plus detector.

### Endogenous IAA measurements

Stems from 5-week-old plants were harvested, after which 2-cm segments from the tip, middle, and base were frozen in liquid nitrogen and ground to a fine powder. Each ground stem segment was mixed with 5 ml extraction medium (methanol:water:formic acid = 15:4:1, v/v/v) containing 5 ng ml^− 1^ deuterium IAA (D_2_-IAA) as an internal standard (Kijidani et al. 2014). After 1-h incubation at 4 °C, 5 ml extraction medium was added and the mixture was stirred with a glass rod before incubating for another 1 h at 4 °C. The mixture was centrifuged at 4000 *g* for 10 min and then the supernatant was collected. The supernatant (10 ml) was purified using the Waters C18 Sep-Pak Cartridge and dried using an evaporator at 60 mmHg and 40 °C. Before loading the supernatant, the cartridge was washed with 5 ml methanol and then equilibrated with 5 ml water adjusted to pH 1.9 with formic acid. The residue dissolved in 4 ml water adjusted to pH 1.9 with formic acid was separated into acidic and basic fractions using the Waters Oasis^®^ MCX Cartridge that was previously washed with 5 ml methanol and then equilibrated with 5 ml water adjusted to pH 1.9 with formic acid. The acidic fraction containing D_2_-IAA was eluted using 4 ml methanol. After filtering the eluates using the Acrodisc^®^ Syringe Filter (PTFE, 13 mm, 0.2 μm), they were concentrated to about 300 µl under decompression. The LC-MS/MS analyses were performed using a Dionex Ultimate 3000 UHPLC system (Thermo Fisher Scientific) equipped with the ACQUITY UPLC® BEH C18 column (1.7 μm, 2.1 × 10 mm) and a Q-Exactive mass spectrometer (Thermo Scientific). The mobile phase comprised solvent A (0.1% formic acid–water, v/v) and solvent B (0.1% formic acid–acetonitrile, v/v). The gradient program was as follows: [time (min)/concentration of A (%)/concentration of B (%)] 0/90/10; 7/30/70; 9/10/90; 23/10/90; 26/90/10; and 30/90/10. The flow rate and injection volume were 300 µl min^− 1^ and 10 µl, respectively.

## Results

### ***CWPO-C*is expressed throughout Arabidopsis and is associated with plant development**

**Fig. 1:**
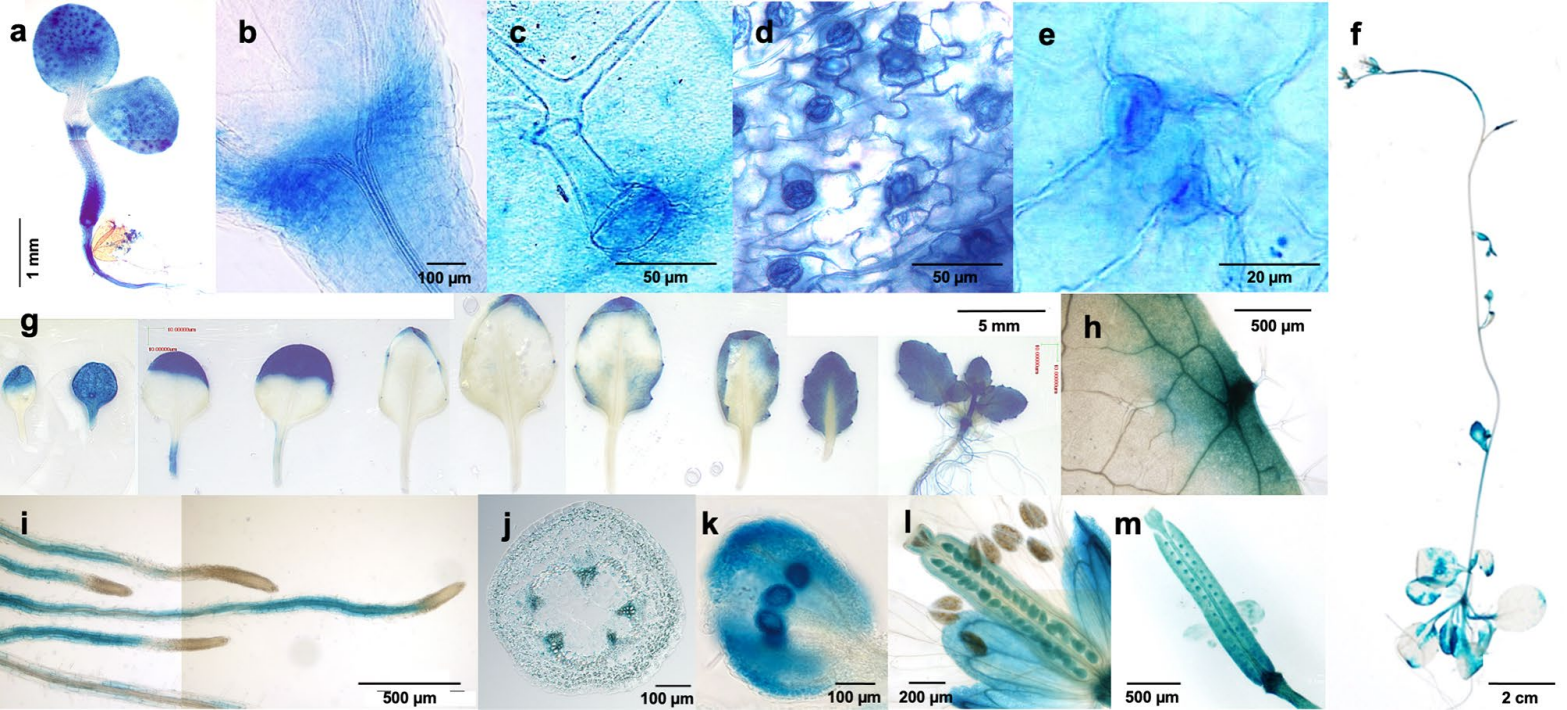
Spatiotemporal expression of *β*-glucuronidase (GUS) driven by the cationic cell-wall-bound peroxidase (*CWPO-C*) promoter in Arabidopsis. **a–e** Analysis of a 2-day-old plantlet. **a** Hypocotyl; **b** Apical meristem; **c** Trichome; **d** and **e** Guard cells. **f** Representative 5-week-old plant. **g** and **h** Leaves from 3-week-old plants. **g** Leaves, left (younger) to right (older); **h** Hydathode. **i** Representative 3-week-old roots. **j** Cross-section of the apical stem of a 6-week-old plant. **k** immature flower. **l** and **m** Representative 6-week-old flower silique. **l** Seed; **m** Silique

A previous RT-qPCR analysis (Sasaki et al. 2006) revealed the *P. alba CWPO-C* gene, which encodes a Class III Prx, is expressed in the xylem, shoot, and leaf. To determine more precisely where and when *CWPO-C* is expressed during plant development, a fusion construct harboring the *GUS* gene under the control of the *CWPO-C* promoter was introduced into Arabidopsis. The staining results indicated *GUS* was expressed in growing organs and tissues that will undergo lignification. The GUS activity was detected in several developing tissues (Fig. 1a–f). More specifically, it was observed in the apical meristem (Fig. 1b) and developing leaves (Fig. 1 g), suggesting that *CWPO-C* is expressed in actively growing and/or differentiating cells. Interestingly, we also detected *GUS* expression in the trichome basal cells (Fig. 1c), in the stomata (mainly guard cells, Fig. 1d, e), and in the hydathodes (Fig. 1h) of the developing leaves, implying that CWPO-C may be important for the differentiation of specific organs. Additionally, GUS activity was detected in the young roots, but not at the root tip (Fig. 1i), in the young vascular bundles in the upper part of the stem (Fig. 1j), in developing seeds (Fig. 1l), immature flowers (Fig. 1k) and in siliques (Fig. 1m), all of which are considered to be undergoing lignification. These observations support our hypothesis that CWPO-C is involved in lignification (Sasaki et al. 2006; Shigeto et al. 2013).

### *CWPO-C* negatively regulates Arabidopsis growth

**Fig. 2:**
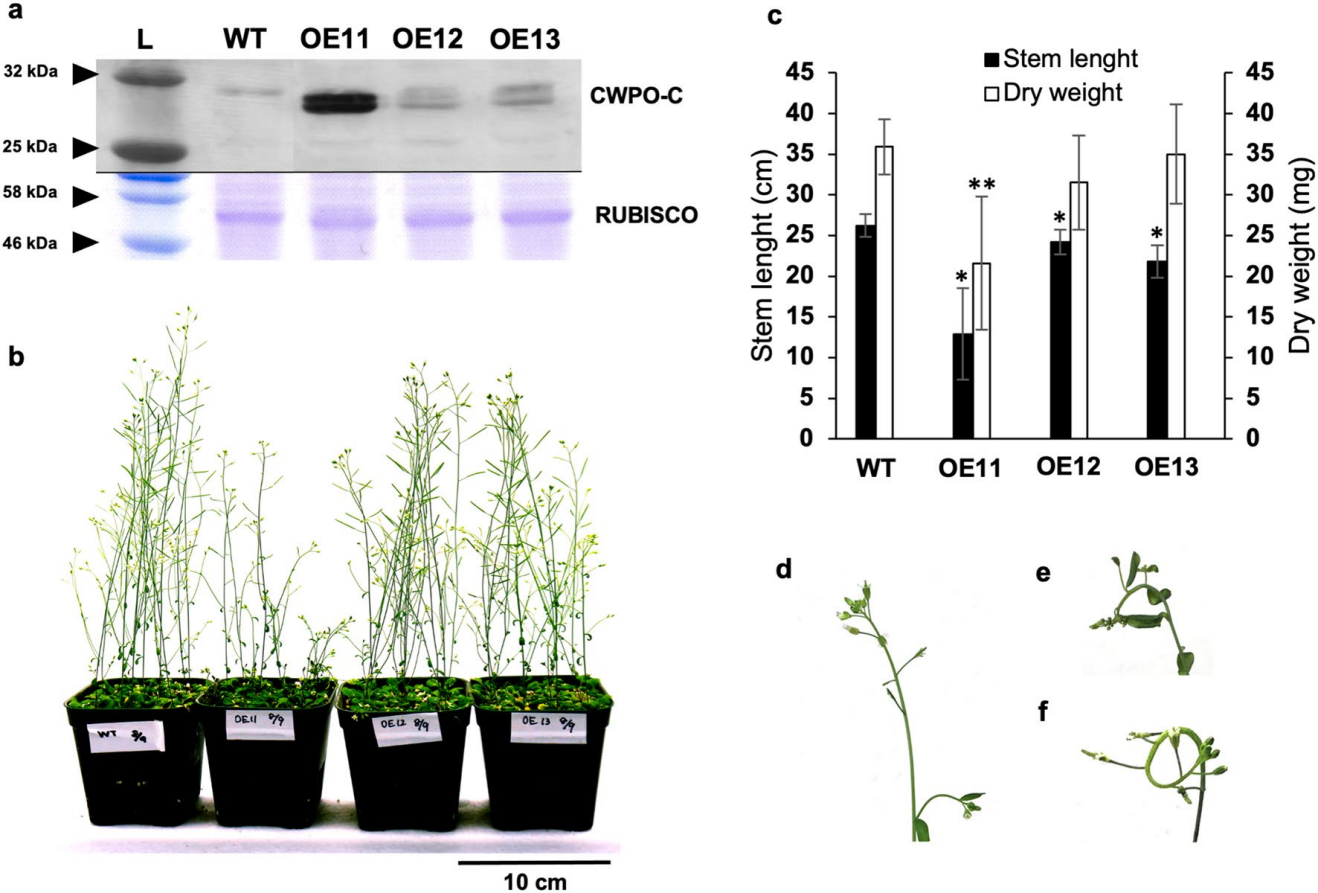
Transgenic *Arabidopsis thaliana* carrying the *P35S*::*CWPO-C* construct. **a** Western blot analysis of CWPO-C-overexpressing transgenic lines 11, 12 and 13 (OE11, OE12, and OE13) and the wild-type (WT) control using an anti-CWPO-C antibody (30 kDa). L: ladder. RUBISCO stained with Coomassie brilliant blue is presented as a reference. **b** Phenotypes of a 6-week-old *P35S*::*CWPO-C* transgenic plant. Dwarfism was characteristic of OE11 plants. Left to right: WT, OE11, OE12 and OE13 **c** Height and dry weight of transgenic lines. Ten biological replicates were analyzed. *: Significant difference (*P* value˂ 0.05) relative to the WT stem length (cm); **: significant difference (*p* ˂ 0.05) relative to the WT dry weight (mg) (Student’s *t* test). **d–f** Apical stem phenotypes of OE11 plants. **d** Straight; **e** Semi-curled; **f** Curled

To functionally characterize CWPO-C in vivo, we generated transgenic Arabidopsis plants expressing *CWPO-C* under the control of the constitutive CaMV 35S promoter. Three of five independent lines of *P35S*::*CWPO-C* (OE11, OE12, and OE13), which expressed *CWPO-C* at different levels according to a western blot analysis of the CWPO-C protein (Fig. 2a, Fig. S2), were subjected to further analyses. Since signals of OE1 and OE9 were weak and similar to OE12, OE1 and OE9 were not considered for further experiments in this study. Among the three transgenic lines OE11, OE12 and OE13, the CWPO-C content was highest in OE11 (Fig. 2a). Compared with the WT plants, all three transgenic lines had shorter stems (Fig. 2b). Line OE11 exhibited particularly poor stem growth (height and dry weight) (Fig. 2c).

Because CWPO-C was most abundant in OE11 plants, this line was characterized further. Interestingly, some OE11 plants exhibited a peculiar phenotypic feature (Fig. 2d–f) during the early stages of bolting, not observed in OE12 neither OE13 plants. Specifically, the OE11 apical stem was semi-curled and/or curled (Fig. 2e, f), with 60% of the plants in successive generations exhibiting this stem trait (Table S1). The OE11 plants with curled and/or semi-curled stems did not produce seeds. The OE11 plants with a straight stem (Fig. 2d) produced small fruits with relatively few seeds. Thus, OE11 plants were always derived from plants without curled stems. Western blot and the subsequent determination of band intensities using ImageJ revealed that the OE11 plants with curled stems contained more CWPO-C protein than the OE11 plants without curled stems (Table S2). Considered together, these findings indicate that high levels of CWPO-C in Arabidopsis negatively affect plant growth, leading to dwarfism and an abnormal curling phenotype. These results imply that CWPO-C influences the Arabidopsis growth mechanism.

### *CWPO-C* overexpression results in a slow stem gravitropic response

**Fig. 3:**
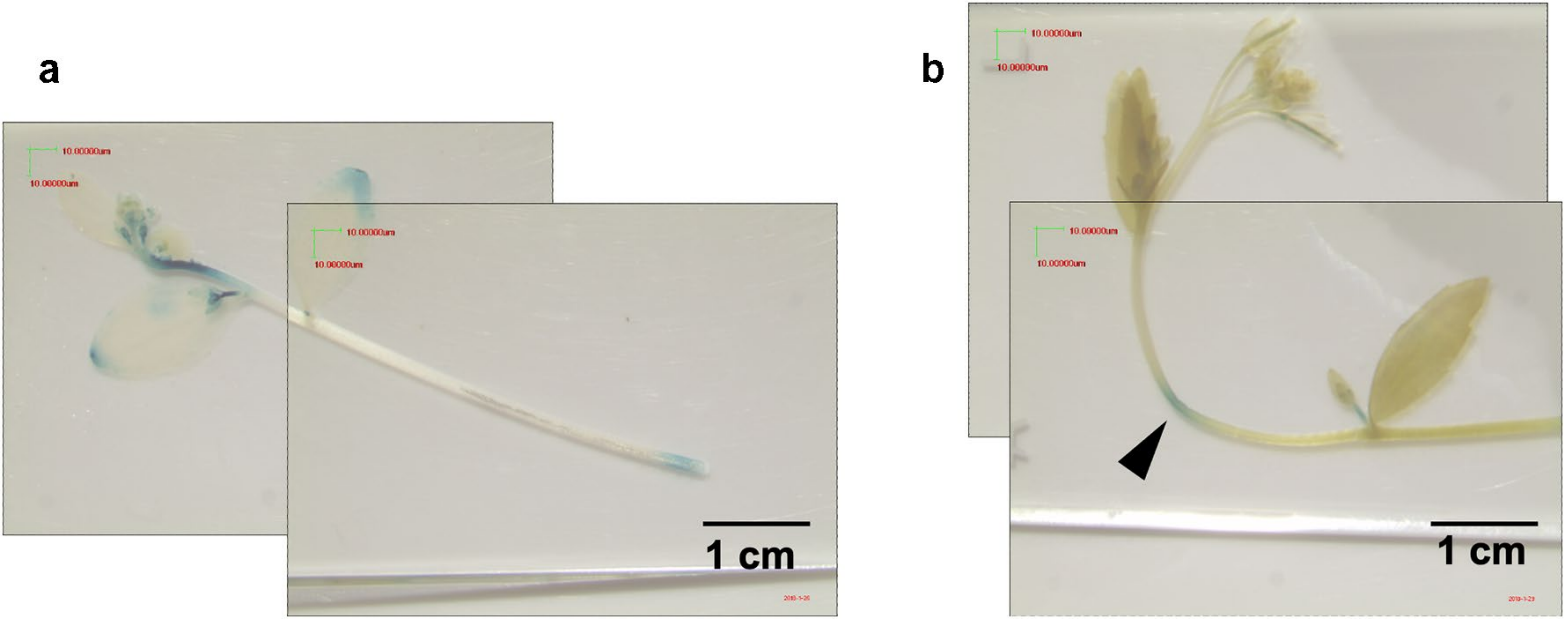
Gravitropic response of the *CWPO-C* promoter in transgenic Arabidopsis plants carrying the *Pcwpo-c*::*GUS* construct. **a** Transgenic plant before bending (control). **b** Transgenic plant analyzed by GUS staining after bending for 120 min. The GUS signal was detected at the bent part of the stem (black arrow)

**Fig. 4:**
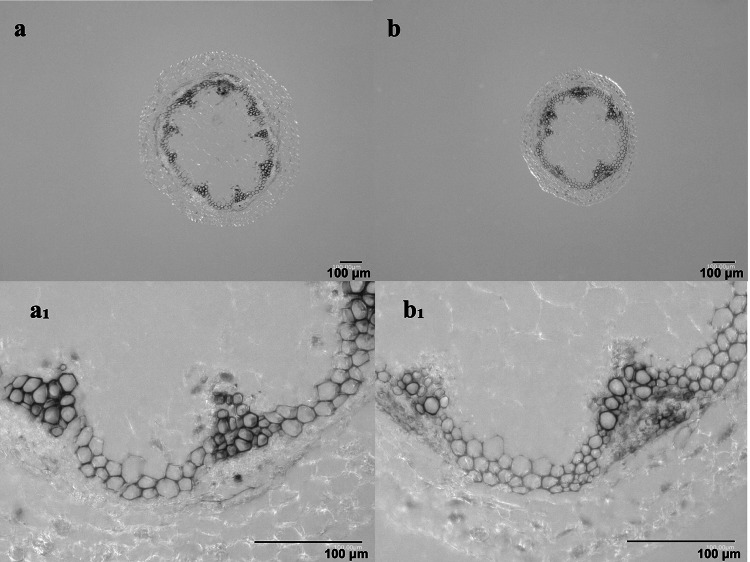
Wiesner staining of 5-week-old Arabidopsis basal stem. **a-a**_1_ WT, **b-b**_1_ OE11

Plant growth and development are regulated by plant hormones (Davies 1995). The observed dwarfism in line OE11 was considered to be related to the plant hormone auxin, which promotes stem elongation and stimulates growth. Modifications in auxin synthesis adversely affect plant growth, ultimately leading to dwarfism (Cheng et al. 2006). Auxin effects in plants were previously evaluated by examining plant responses to a gravitropic stimulus (Salisbury et al. 1988). In the present study, time taken to reach a 90° stem curvature in OE11, OE12, OE13 and WT Arabidopsis plants were set horizontally was recorded. OE plants bent more slowly and the time required for the stems to bend 90° was almost double that required by WT plants (Table 1). In addition, Wiesner staining observations of the cell walls showed very similar between WT and OE11 (Fig. 4), suggesting that cell wall and lignification levels may not affect largely to the delay of the bending time in OE11. These results suggest the possibility that *CWPO-C* overexpression modulated the gravitropic response by disrupting auxin accumulation in plant. When *Pcwpo-c*::*GUS* transgenic plants were subjected to the bending test, GUS activity was clearly induced in the bended part of the stem, after 120 min of bending (Fig. 3b). While, at zero time of bending test, *Pcwpo-c*::*GUS* transgenic plant expressed typical GUS activity in the apical stem, young leaves and flowers (Figs. 3a and 1b, g, l respectively), but after 120 min of bending GUS expression in these organs disappeared for unknown reasons. These observations suggest that CWPO-C affects auxin concentration, thereby altering the stem gravitropism response.

**Fig. 5:**
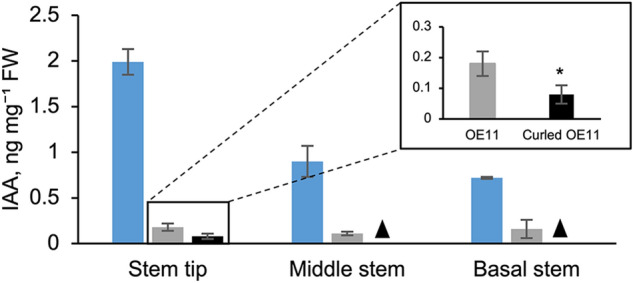
Analysis of the IAA content in the stems of 5-week-old WT (blue), OE11 (gray), and curled OE11 (black) plants. Data are presented as the mean ± standard deviation. Three biological replicates were analyzed. *: Significant difference (*P* value ˂ 0.05) relative to the OE11 stem tip (Student’s *t* test). Filled triangle: IAA contents in the middle and basal parts of the curled stem were not determined because the samples were too small (colour figure online)

**Table 1:** Bending time of 4-week-old wild-type and OE11-13 Arabidopsis plants

Type	Bending time (min)
Wild-type	86 ± 4.1
OE11	150 ± 3.5
OE12	152 ± 4.1
OE13	132 ±14.4

Bending time refers to the time required for the horizontally placed stem to bend 90°. Four biological replicates were analyzed and the data are presented as the mean ± standard deviation. OE11-13: *CWPO-C*-overexpressing line 11–13

### Quantification of IAA in Arabidopsis overexpressing *CWPO-C*

If CWPO-C has an effect on auxin, the auxin content should differ between OE11 and WT plants. According to OE11 results, such higher CWPO-C protein content, shortest stem length and presence of characteristic curled stem phenotype in comparison to OE12 and OE13 lines, we decided to quantify IAA content only in OE11, since it was the most representative line of CWPO-C effects over IAA related phenotype. Stems from 5-week-old WT and OE11 plants were collected. Regarding line OE11, the straight and curled stems were collected separately. The stems were divided into upper, middle, and lower parts for an analysis of the IAA content (Fig. 5). In the stem tip, the IAA content was approximately 10-times lower in OE11 plants than in WT plants. Regarding the other stem segments, the IAA contents in the middle and lower parts were lower in OE11 plants than in WT plants (Fig. 5). Furthermore, the IAA content in the curled stem tip of OE11 plants was only about half of that in the straight stem tip of OE11 plants (Fig. 5). The dwarfism and curled stem phenotype of OE11 may be attributed to a decrease in IAA contents. The overexpression of *CWPO-C* appears to decrease IAA levels in OE11 plants with or without curled stems. The in vitro reaction, in which rCWPO-C reacted and decreased IAA (Fig. S1), supported the observed IAA decrease in OE11.

## Discussion

Earlier research indicated CWPO-C has a versatile oxidizing activity that is unaffected by the substrate size because the oxidation site is exposed on the protein surface (Shigeto et al. 2012), similar to fungal lignin-degrading Prxs (Doyle et al. 1998; Miki et al. 2011; Ruiz-Dueñas et al. 2009, 2009). Additionally, CWPO-C may contribute to lignification because of its rare activity (i.e., ability to oxidize sinapyl alcohol and high molecular weight lignin polymers) (Aoyama et al. 2002; Sasaki et al. 2004). This hypothesis was proposed on the basis of in vitro biochemical experiments (Sasaki et al. 2006, 2008); however, there was no direct biological evidence confirming the hypothesis. Lignin provides structural support and is responsible for the hydrophobic properties of water-conducting cells, especially in the xylem. Therefore, the gene encoding a lignification-related Prx is expected to be expressed around tissues in which lignin is being deposited, including xylem and interfascicular fibers. The results of the current study indicated that *CWPO-C* is expressed in the roots, xylem, and silique (Fig. 1i, j, m, respectively). This is consistent with the possibility CWPO-C catalyzes lignification. However, *CWPO-C* was also expressed in immature tissues, such as meristems, young upper stems, leaves, flowers, and fruits (Fig. 1b, f, g, k and m, respectively). A real time polymerase chain reaction (RT-qPCR) analysis was performed in *Populus alba* L., and the results showed *CWPO-C* expression in shoot, xylem, and leaf (Sasaki et al. 2006), similar to transgenic *Pcwpo-c*::*GUS* Arabidopsis.

Among the transgenic lines overexpressing *CWPO-C*, OE11 had the highest CWPO-C content as well as curled stems, which was an unexpected phenotype. Although the OE11 plants used in this study were homozygous, there were differences in the CWPO-C content between individual plants. A western blot analysis of the OE11 plants with and without curled stems revealed that the amount of CWPO-C was about 1.3-times greater in the curled stem tip than in the straight stem tip (Table S2). Thus, an excessive amount of CWPO-C may cause stem tips to curl and lead to sterility. Gravitropism causes stems to bend because of the associated change in auxin distribution. The time required for a stem placed horizontally to bend 90° was used as a simple index, but sensitive assay, for investigating whether auxin is involved in the stem curvature observed in CWPO-C OE plants. A significant delay in the bending of the OE11, OE12 and OE13 stem (Table 1) suggests that CWPO-C interferes with the plant cell elongation mechanism. Regarding the gravitropic response, OE11 and WT showed similar cell wall lignin in the Wiesner staining (Fig. 4), which let us deduce that the delay in bending time of OE11 was not due to changes in the cell wall lignin. Rakusová et al. (2011) observed that inhibiting the auxin transporter PIN3 leads to decreased auxin mobilization during gravitropism, which results in delayed bending and a smaller curvature. In another study, Gutjahr et al. (2005) demonstrated that an IAA treatment increases the curvature angle and accelerates the bending of rice hypocotyls. The inhibition of stem bending in the current study may be explained by the inactivation of auxin through the oxidation by CWPO-C. Furthermore, the disrupted IAA distribution due to an excess of CWPO-C is a plausible explanation for the aforementioned curled stem tip of OE11 plants. It is also possible that the stem curvature in OE11 plants was the result of Prx-derived •OH radicals exhibiting an auxin-like effect. Peroxidases help strengthen cell walls via lignification, but they can also weaken cell walls and promote cell elongation, with the Prx-derived •OH inducing the oxidative cleavage of cell wall polysaccharides (Liszkay et al. 2003; Kunieda et al. 2013).

In this study, OE11 plants had decreased IAA contents (Fig. 5). Moreover, the severely curled OE11 stem tips contained more CWPO-C and significantly less IAA than the straight OE11 stem tips (Table S2, Fig. 5 respectively). The observations described only on OE11 line still have a low possibility that IAA decrease can be due to the insertion site of the transgene but not the peroxidase itself. On the other hand, the similar phenotype such the reduced stem length and bending time, observed in all three lines (OE11, OE12 and OE13), might be due to the peroxidase expression. This suggests that CWPO-C catabolizes IAA in plants. The reaction of IAA with Class III Prxs, such as horseradish Prx, were first demonstrated under in vitro conditions (Galston et al. 1953). The PxB2 Prx from *Vitis vinifera* roots was reported to have oxidase activity toward IAA (Vatulescu et al. 2004). In a preliminary assay, rCWPO-C reacted with IAA in vitro, the results showed 2 unidentified oxidative products of IAA, demonstrating the oxidative activity of rCWPO-C towards IAA (Fig. S1). We had also confirmed that two major products are not 2-oxindole-3-acetic acid (oxIAA) in the rCWPO-C - IAA in vitro reaction (Fig. S1). It seems that IAA oxidation by rCWPO-C differs from previous reported oxidation pathway, where oxIAA is the major oxidation product. There have been reports describing the Prxs in IAA reaction in vitro; however, the available information regarding Prx-catalyzed IAA *in planta* is very limited. It has been well-known that some peroxidases expression could be affected by other peroxidases (Cosio et al. 2009), therefore, it is difficult to attribute the peroxidase activity changes only to the overexpression of the peroxidase introduced. For this reason, we focused on the effect of *CWPO-C* overexpression to the phenotype in this study. Previously, Cosio et al. (2009) proved that the heterologous expression of *CpPrx01* from *Cucurbita pepo* in Arabidopsis lengthens the roots and hypocotyls and decrease the IAA levels. In contrast, the OE11 plants in this study had shortened stems (Fig. 2b) and decreased IAA levels (Fig. 5), which is similar to the auxin-deficient phenotype of Arabidopsis mutants in which multiple *YUC* genes are silenced (Cheng et al. 2006; Chen et al. 2014), however, there have been no report that described the direct relation between peroxidase and *YUC* expression, so far. The results of the earlier study by Cosio et al. (2009) are not inconsistent with our findings because auxin effects on cell elongation (i.e., promotion or suppression) depend on the concentration and the organ (Tanaka et al. 2006; Vanneste and Friml 2009). In fact, compared with the WT plants, the OE11, OE12, OE13 lines had shorter stems, but longer roots (Table S3).

## Conclusion

Our results demonstrated that the overexpression of *CWPO-C*, which encodes a class III peroxidase, decreases the IAA content in Arabidopsis and results in the phenotype that is consistent with the typical phenotype due to the auxin deficiency. This strongly suggests that CWPO-C participates in the regulation of plant growth and affects IAA concentration. Furthermore, CWPO-C may play a role in the termination of cell elongation, rather than controlling the initiation of these processes via auxin.

## Electronic supplementary material

Below is the link to the electronic supplementary material.

Supplementary file1 (PDF 749 kb)
