# Correction to: *Populus alba* cationic cell-wall-bound peroxidase (CWPO-C) regulates the plant growth and affects auxin concentration in *Arabidopsis thaliana*

**DOI:** 10.1007/s12298-022-01259-4

**Published:** 2022-12-16

**Authors:** Diego Alonso Yoshikay-Benitez, Yusuke Yokoyama, Kaori Ohira, Koki Fujita, Azusa Tomiie, Yoshio Kijidani, Jun Shigeto, Yuji Tsutsumi

**Affiliations:** 1 Department of Agro-environmental Sciences, Graduate School of Agriculture, Kyushu University, 744 Motooka Nishi-ku, Fukuoka, 819-0395 Japan; 2 Faculty of Agriculture, Kyushu University, 744 Motooka Nishi-ku, Fukuoka, 819-0395 Japan; 3 Division of Forest and Environmental Science, Faculty of Agriculture, University of Miyazaki, 1-1 Gakuen Kibana-dai Nishi, Miyazaki, 889-2192 Japan; 4 Office of Research and Academia Government Community Collaboration, Hiroshima University, 1-3-2 Kagamiyama, Higashihiroshima, Hiroshima 739-8511 Japan

**Correction to: Physiol Mol Biol Plants (September 2022) 28(9):1671–1680** https://doi.org/10.1007/s12298-022-01241-0

In this article the family name of the fifth author Tomiie was incorrectly written as Tomie.

The correct name should read as Azusa Tomiie.

The original article has been corrected.

