# Supplementary material for: Populus alba cationic cell-wall-bound peroxidase (CWPO-C) regulates the plant growth and affects auxin concentration in Arabidopsis thaliana
Source: Physiol Mol Biol Plants. 2022 Oct 30;28(9):1671–80. doi: 10.1007/s12298-022-01241-0 (PMC9636347; doi:10.1007/s12298-022-01241-0)
Supplement: Supplementary file 1 — Supplementary file1 (PDF 749 kb) [file 12298_2022_1241_MOESM1_ESM.pdf]

***Populus alba*** **cationic cell-wall-bound peroxidase (CWPO-C) regulates the plant growth and affects auxin concentration in *Arabidopsis thaliana***

*Physiology and Molecular Biology of Plants*

**Supplementary information**

**Table S1.** Proportion of OE11 plants with a curled phenotype in successive generations

| Generation | Plants with straight stems | Plants with curled stems | Total number of plants | Curled (%) |
|---|---|---|---|---|
| 1st | 9 | 15 | 24 | 63 |
| 2nd | 13 | 14 | 27 | 52 |
| 3rd | 19 | 18 | 37 | 49 |
| 4th | 12 | 20 | 32 | 63 |
| 5th | 7 | 11 | 18 | 61 |

Note: The 1st generation is the $T_6$ generation, which was confirmed as homozygous.

**Table S2.** Integrated band intensity of CWPO-C in the stem tip of OE11 and curled OE11 plants and the relative band intensity of CWPO-C normalized against the intensity of RUBISCO

| Phenotype | Average integrated band intensity | Relative integrated band intensity |
|---|---|---|
| Curled stem | 13,595 ± 508 | 1.31 ± 0.04 |
| Straight stem | 9,638 ± 172 | 0.97 ± 0.04 |

Note: The band intensities for the extracted proteins were determined for three biological replicates per phenotype. Data are presented as the mean ± standard deviation.

**Table S3.** Development of 6-week-old *CWPO-C*-overexpressing transgenic and wild-type (WT) Arabidopsis plants

| Line | Stem length (cm) | Dry weight (mg plant$^{-1}$) | Root length (cm) | Number of lateral branches per plant | Number of rosette leaves per plant |
|---|---|---|---|---|---|
| OE11 | 12.9 ± 5.6* | 21.6 ± 8.2* | 8.0 ± 1.0* | 3.4 ± 0.5 | 10.9 ± 1.3 |
| OE12 | 24.2 ± 1.5* | 31.5 ± 5.8 | 7.6 ± 1.2* | 2.5 ± 0.5 | 11.1 ± 1.8 |
| OE13 | 21.8 ± 2.0* | 35.0 ± 6.1 | 7.1 ± 1.2* | 3.3 ± 1.1 | 12.3 ± 1.8 |
| WT | 26.2 ± 1.4 | 35.9 ± 3.4 | 5.8 ± 1.4 | 3.2 ± 0.6 | 11.0 ± 1.3 |

Note: Data are presented as the mean ± standard deviation (n = 10). Asterisks indicate significant differences between the OE lines and the WT control (*, $p < 0.03$, Student's *t* test).

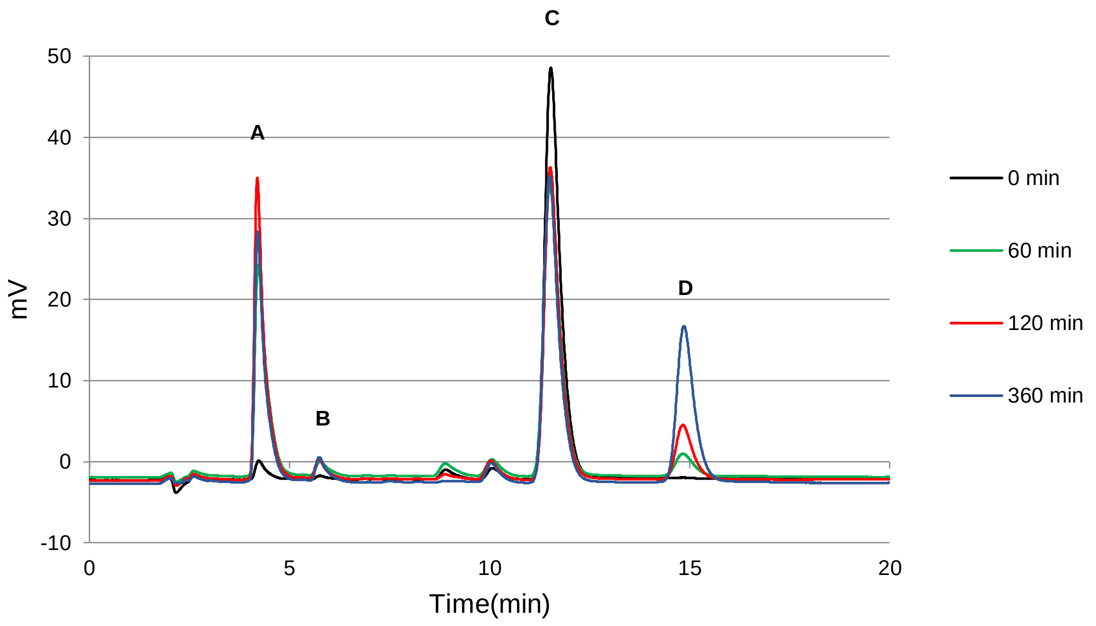


**Fig. S1** Elution profiles of the oxidative products of IAA by recombinant CWPO-C. The HPLC chromatograms at the reaction time 0, 60, 120, 360 min were shown. A: 4.24 min product peak, B: 5.75 min oxIAA peak, C: 11.53 min IAA peak and D: 14.86 min product peak. The reaction products were analyzed on the reverse-phase HPLC with a AQ-$C_{18}$ InertSustain column. The eluted products were monitored at O.D. 250 nm

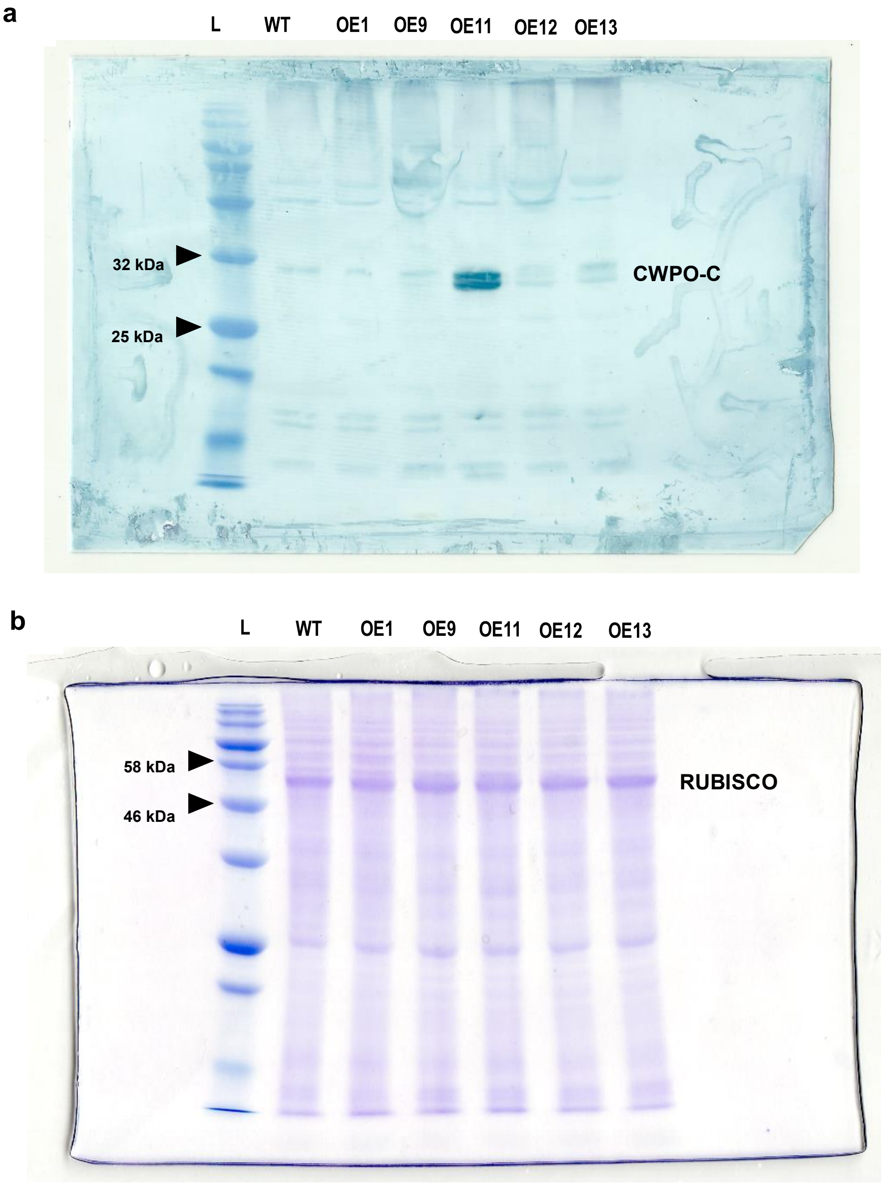


**Fig. S2** Original gel images of, western blot and CBB analysis (**a** and **b**, respectively), of CWPO-C-overexpressing transgenic lines 1, 9, 11, 12 and 13 (OE1, OE9, OE11, OE12, and OE13) and the wild-type (WT) control using an anti-CWPO-C antibody (30 kDa). L: ladder. RUBISCO stained with Coomassie brilliant blue is presented as a reference

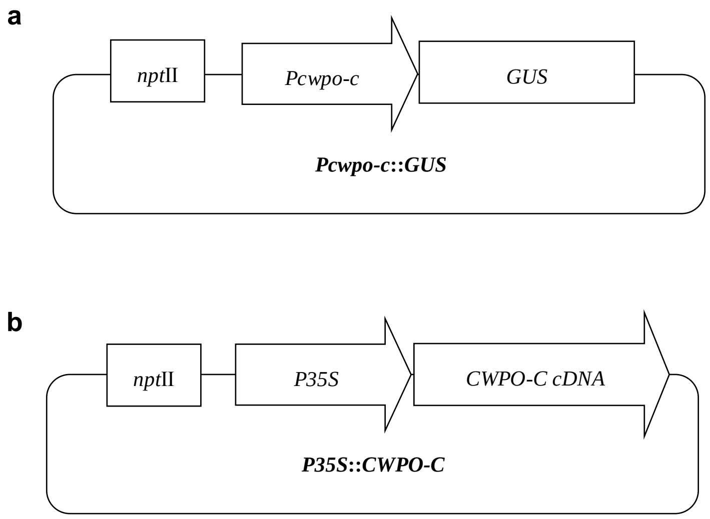


**Fig. S3** Diagram of construct details for, *Pcwpo-c*::*GUS* and *P35S*::*CWPO-C* (**a** and **b**, respectively)
